# ‘Fetal side’ of the placenta: anatomical mis-annotation of carbon particle ‘transfer’ across the human placenta

**DOI:** 10.1038/s41467-021-26437-y

**Published:** 2021-12-03

**Authors:** Beth Holder, John D. Aplin, Nardhy Gomez-Lopez, Alexander E. P. Heazell, Joanna L. James, Carolyn J. P. Jones, Helen Jones, Rohan M. Lewis, Gil Mor, Claire T. Roberts, Sarah A. Robertson, Ana C. Zenclussen

**Affiliations:** 1 Institute of Reproductive and Developmental Biology, Department of Metabolism, Digestion and Reproduction, Imperial College London, London, UK; 2 Maternal and Fetal Health Research Centre, School of Medical Sciences, University of Manchester, Manchester Academic Health Sciences Centre, St Mary’s Hospital, Manchester, UK; 3 Perinatology Research Branch, Division of Obstetrics and Maternal-Fetal Medicine, Division of Intramural Research, National Institute of Child Health and Human Development, National Institutes of Health, U.S. Department of Health and Human Services Detroit, MI, USA; 4 Department of Obstetrics and Gynaecology, School of Medicine, FMHS, University of Auckland, Auckland, New Zealand; 5 Departments of Physiology and Functional Genomics & Obstetrics and Gynecology, College of Medicine, University of Florida, Gainesville, FL USA; 6 The University of Southampton, Southampton, UK; 7 Wayne State University School of Medicine, Detroit, MI USA; 8 Flinders Health and Medical Research Institute, Flinders University, Bedford Park, SA Australia; 9 The University of Adelaide, Adelaide, SA Australia; 10 Department of Environmental Immunology, Helmholtz Centre for Environmental Research, Leipzig, Germany

**Keywords:** Cell biology, Anatomy

**arising from** H. Bové et al. *Nature Communications* 10.1038/s41467-019-11654-3 (2019)

In utero exposure to environmental agents is a critical driver of diseases manifesting in childhood and adulthood^1,2^, and direct fetal contact with potentially harmful substances is largely determined by the ability of the material to cross the placenta, which forms a selective barrier between maternal and fetal circulations. In a recent *Nature Communications* article, Bové et al. present data which they report as demonstrating that ‘ambient black carbon particles reach the fetal side of human placenta’^3^, which centres on their demonstration that carbon particles are detectable in placental villous tissue, as visualised by two-photon microscopy. Most people will interpret the term ‘fetal side’ to mean that particles have moved from the maternal circulation into cells adjacent to the fetal circulation, yet, the data presented shows that carbon particles are contained in the placental villous tissue, and does not demonstrate transfer to the fetal side. The data presented are interesting, and the techniques used a valid way of studying entry of carbon particles into tissues, yet the conclusion as stated in the title is open to misinterpretation, which was strongly evident in media coverage of this publication.

Whether carbon particles, or other pollutants, can cross the placenta to the fetus is an important question to investigate. The images presented in Bové et al., show the presence of carbon particles primarily in trophoblast, the outermost cellular layer that contacts maternal blood, indicating that particles are likely trapped in the maternally-facing side of the placenta. Villous tissue was sampled at different depths within the placenta. However, increased distance from the maternal attachment site does not correspond to greater risk of fetal exposure, as even in deeper locations the particles are still within the trophoblast layer in contact with maternal blood and are protected from the fetal circulation. It is also very important that samples of maternal decidua are not confused as being placental tissue. Here, we argue the importance of employing accurate terminology in studies of the maternal-fetal interface to avoid misinterpretation of research findings. Indeed, it can be argued that there *is* no ‘fetal side’ of the placenta, as the human placenta is, in its entirety, a fetal organ.

To understand transfer of substances from mother to fetus, it is vital to appreciate the complex structure of the placenta, which enables maternal and fetal blood to come into close proximity, whilst maintaining physical separation. Figure 1a illustrates a cross-section of the human placenta at term. The functional area for exchange of gases and nutrients is the chorionic villous tissue, containing placental blood vessels that connect directly into the fetal circulation via the umbilical cord. These villi are bathed in maternal blood within the intervillous space. The maternal endometrium at the site of placental attachment undergoes remodelling, forming the decidua basalis. At birth, the placenta is delivered with an attached layer of maternal decidua, enabling the study of both placental and decidual tissues ex vivo. The different cellular make-up and structure of these two tissue types can be seen in Fig. 1b.

**Fig. 1:**
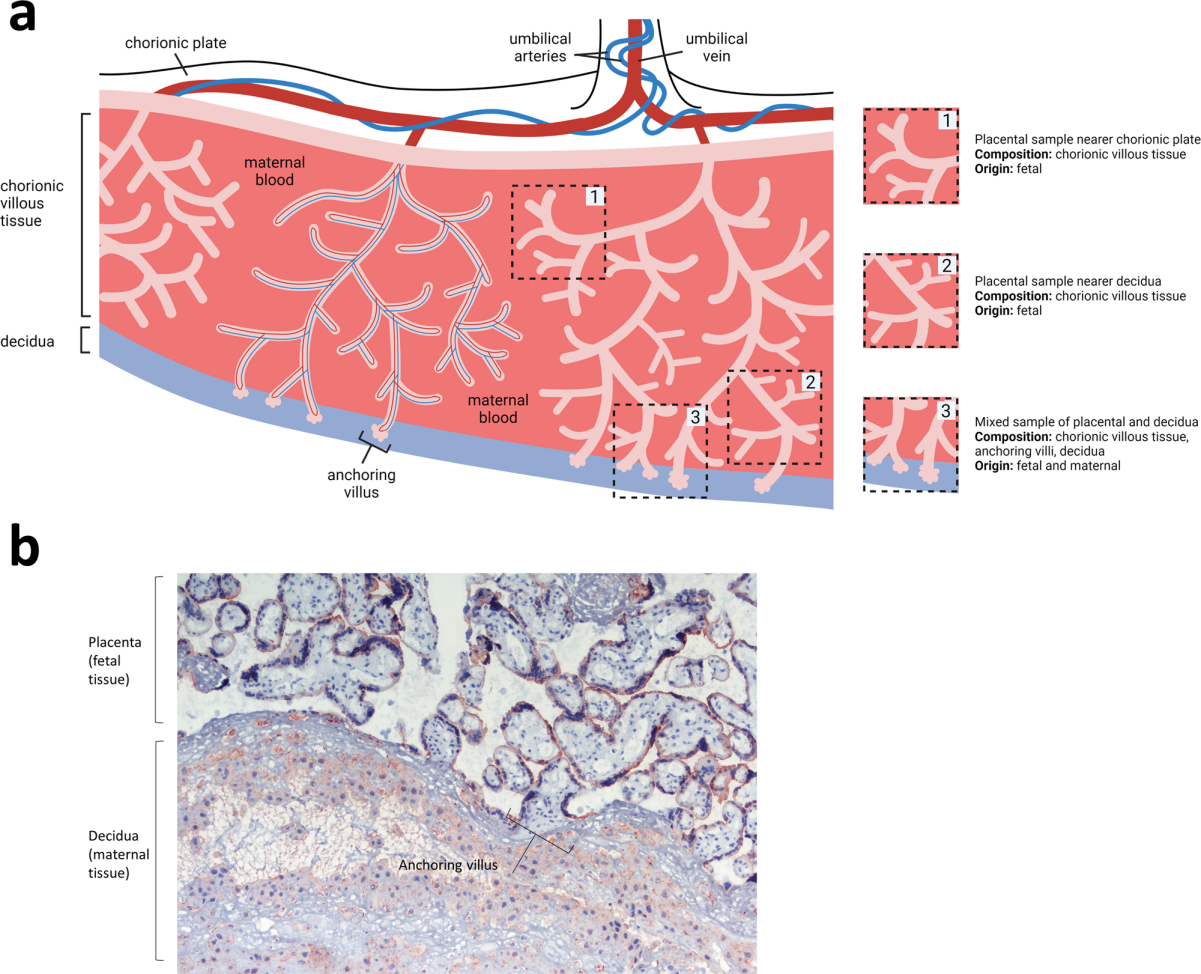
Structure of the term placenta, and biopsy sampling. **a** The umbilical vein and arteries containing fetal blood reach the placenta at the cord insertion site and spread across the chorionic plate, branching extensively to provide the tree-like shape of the placenta. The branched vessels are embedded in a stromal core covered by trophoblast cells which are completely bathed in maternal blood within the intervillous space. Anchoring villi attach the placenta to the maternal endometrium (decidua). The three dashed-line boxes indicate the potential biopsy locations utilised by Bove et al. (1) and (2) are standard placental biopsies, comprising chorionic villous tissue that is fetal in origin, whilst excluding contamination with maternal decidual tissue. Regardless of whether a placental biopsy is taken near to the chorionic plate (1) or close to the decidua (2), the tissue is totally fetal. (3) represents an alternative possible location for the ‘maternal side’ biopsy employed by Bové et al. which is a mixed tissue biopsy comprising fetal chorionic villous tissue and anchoring villi, and a layer of maternal decidual tissue. Created with Biorender.com. **b** Micrograph of term maternal-fetal interface demonstrating the different structure of the decidua layer (maternal) that is delivered with the placenta (fetal). Decidua contains a mix of stromal cells, leucocytes and invaded extravillous trophoblast. Tissue stained with antibodies to Cytokeratin 7, which marks placental trophoblast. Nuclei counterstained with haematoxylin.

Sampling location is central to the study by Bové et al., as it is important to know whether the tissue contains fetal and/or maternal components, but it is not well described, and unorthodox definitions have been utilised to describe the sites of biopsies. Bové et al. take what they term a ‘fetal’ biopsy by excising chunks of villous tissue underneath the chorionic plate. Box 1 in Fig. 1a shows our interpretation of the location of this biopsy, which is a standard placental biopsy of chorionic villous tissue. The nature of the ‘maternal’ biopsy is more ambiguous, being described as taken at the ‘side facing towards…the mother’. This could describe the location indicated by box 2 in Fig. 1a; which would be the same in composition as the ‘fetal’ biopsy indicated by box 1. Alternatively, it could describe the location shown in box 3 of Fig. 1a, which would yield a mixed biopsy of fetal placental tissue and maternal decidua basalis. Either approach is fine, if combined with the correct terminology, rather than the terminology used in this paper. A chorionic villous sample, taken nearer the decidua (box 2) is not the ‘maternal side of the placenta’; it is a placental sample, the same as a sample taken a bit nearer the chorion (box 1). A mixed biopsy of placenta and decidua (box 3) is also not the ‘maternal side of the placenta’, in the way it is being interpreted in this study. The importance of consistent sampling of placentas, and methods for achieving this, is reviewed by Burton et al.^4^.

The word decidua does not appear in this paper, although we think that this is probably what is being examined in the ‘maternal biopsies’. Unfortunately, although summary data of particles within the biopsies are provided in bar graphs (Supplementary Fig. 4), Bové et al. do not show any images of the ‘maternal biopsies’, so it is not possible to determine what tissue/cells are examined, and whether particles are in placental tissue or in decidua (as shown by us in Fig. 1b). This is central to the interpretation of this study, so it is concerning that these details are not reported. The lower particle count reported on the ‘maternal side’ of the placenta could be due to microscopy being performed on a completely different tissue; the decidua basalis.

Once an understanding of the gross anatomy of the human placenta is recognised, transfer studies must also consider the complex cellular organisation of the placental villus, the major site of exchange between maternal and fetal circulations (Fig. 2). The floating villi are covered in a multinucleated syncytium in direct contact with maternal blood. Underneath this lie the cytotrophoblast cells and the inner stromal core, containing placental macrophages, fibroblasts, and a capillary endothelium containing fetal blood^5^. Thus, for particles to move from maternal to fetal circulations, they must cross several cell layers and basement membranes. All these cell types arise from the blastocyst, and are therefore fetal in origin. Although fundamental to the interpretation of particle localisation, none of these cell types are mentioned in this study.

**Fig. 2:**
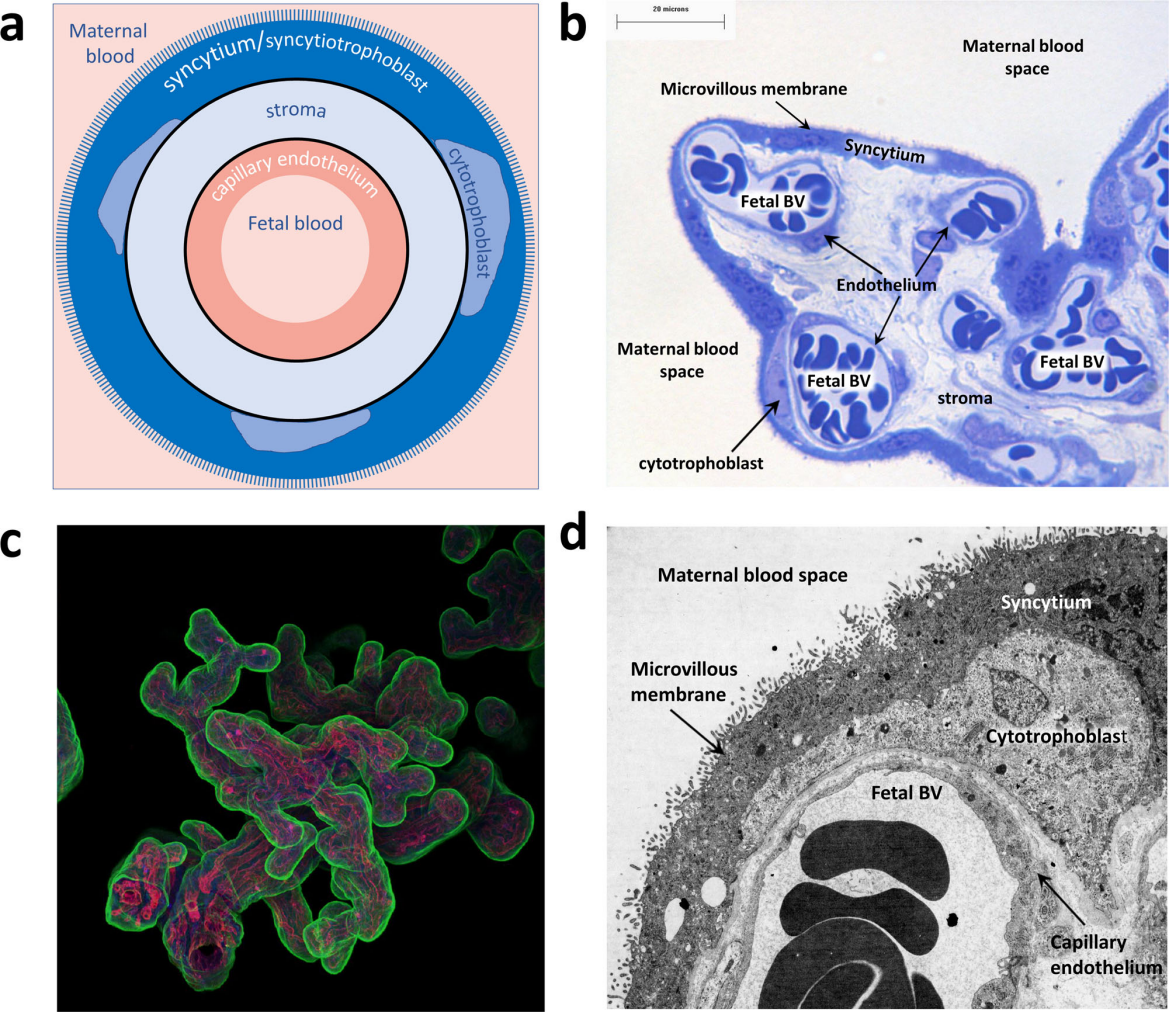
Cellular organisation and structure of the human placenta, demonstrating the cell layers between maternal and fetal circulations. The surface of the placenta facing the maternal blood is covered by a multinucleate syncytiotrophoblast with a microvillous surface to facilitate exchange. The syncytiotrophoblast layer is formed by the fusion of the underlying cytotrophoblast cells. Beneath this lies the stroma, which contains fibroblasts and placental macrophages, and placental blood vessels lined by endothelial cells. **a** Diagram of a cross-section through a placental villus, showing the fetal syncytiotrophoblast in contact with maternal blood and fetal blood in the capillary endothelium. **b** Semi-thin section of term placenta stained with Toluidine blue. Fetal BV; fetal blood vessel. **c** Whole-mount term placenta stained with lectins illustrating the syncytiotrophoblast in contact with maternal blood in green, stroma in blue and the fetal capillaries in magenta. **d** Electron micrograph illustrating the three main cell layers between the maternal and fetal circulations.

A micrograph presented by Bové et al. shows a cross-section of term placental villous tissue familiar to placental biologists. Carbon particles are visible within this tissue (reported as the ‘fetal side’). Unfortunately, the magnification and resolution are too low to precisely identify the particle location. Nevertheless, it appears most particles are lodged in the syncytiotrophoblast, the first barrier layer of the placenta. The syncytiotrophoblast is highly endocytic and constantly samples its environment through fluid phase endocytosis^6^. It is, therefore, not surprising that carbon particles could be taken up into this cell layer, if they are present in the maternal circulation. Critically, the syncytiotrophoblast is an important barrier, trapping harmful components to prevent transfer to the fetus. Hence, the presence of particles in the syncytium does not demonstrate transfer per se. This is exemplified by a recent study^7^ where macrophage-enriched placental cells were examined for ingested carbon particles; the bulk of these, when visualised by transmission electron microscopy, were of trophoblast origin as can be attested by one of us (CJ) who analysed the cell samples. This is not to say that such ingestion does not have deleterious effects, as there is evidence of reduced cellular growth and altered protein expression of first trimester trophoblast cultured with urban pollution particles^8^. The trophoblast and syncytiotrophoblast are not mentioned in the interpretation of the results.

To definitively demonstrate penetration of the placental barrier, certain criteria must be met. Specifically, higher magnification images are required to enable proper visualisation of the location of particles within cell layers other than the syncytiotrophoblast such as the stroma or capillary endothelium; regions that are not mentioned in the entirety of the paper. The interpretation of potential transfer across the placental barrier towards the fetus would be justified if particles were clearly demonstrated to have crossed the syncytiotrophoblast and to be present within the villous stroma and/or the endothelium of fetal blood vessels (or in cord blood), substantiated by co-localisation of cell lineage-specific markers.

It could alternatively be argued that Bové et al. have shown the presence of carbon particles in the villous stroma; potentially there are two particles located either on the basal side of cytotrophoblast cells or within the stroma in Fig. 2 (red and blue inset boxes). It is very hard to judge this, as the stroma is not mentioned anywhere in the paper, and the images presented are not of sufficient magnification nor resolution for the reader to determine this localisation themselves. Supplementary Fig. 2a shows carbon particles inside a cell that does not appear to be trophoblast, which is intriguing. The authors describe this location as ‘inside the placental tissue’. Unfortunately, it is not stated what cell type it is. It is certainly not trophoblast, nor is it endothelial, as it has no basal lamina. It does not resemble a fibroblast nor a macrophage. Without more details of its location, it is hard to interpret this data. Likewise, Supplementary Fig. 2b shows aggregates inside some type of cell, but it is not possible to conclude what cell type this is and where the particles are located within the placenta without the provision of some orientating information.

Growing evidence points to a link between maternal exposure to environmental pollutants during pregnancy and adverse effects on fetal and childhood health^9–13^. However, the negative effects observed in offspring after perinatal/in utero exposure to environmental contaminants do not necessarily indicate that the pollutants reached the fetus, as indirect effects via the mother and the placenta could be responsible. The study by Bové et al. nicely supports previous suggestions that carbon particles can be taken up from maternal blood into the human placenta. However, the information presented to the reader does not demonstrate maternal to ‘fetal side’ transfer, although we don’t rule out that this is possible. The terminology employed in this article is not in common usage in the academic community, and misrepresents the structure and function of the human placenta and nature of the feto-maternal interface.

Similar inaccurate ‘fetal side’ terminology has been utilised in other recent papers on the topics of placental uptake/transfer of SARS-COV-2^14^ and microplastics^15^. Worryingly, these misinterpretations are further amplified in the media, as there is strong public interest in studies with implications for fetal and infant health. More definitive reporting of the tissue composition of the biopsies analysed, as well as higher resolution/magnification images that enable accurate localisation of the carbon particles within precise anatomical sites, are necessary to support a convincing case for carbon particle transfer across the human placenta. Only with both a sufficiently rigorous technical approach, clearly presented data and accurate interpretation can we reliably advance understanding of the environmental determinants of pregnancy outcome and offspring health.

## Methods

### Human subjects

Where required, we have complied with all relevant ethical regulations, and informed consent was obtained from participants. Ethical approval was provided by the Northern X Regional Ethics Committee (New Zealand; NTX/12/06/057/AM09) or Southampton and Southwest Hampshire Local Research Ethics Committee (UK; 11/SC/0529).

### Immunohistochemistry

Paraformaldehyde-fixed term human placenta-decidua biopsies were snap frozen in OCT compound, cut and subjected to heat induced antigen retrieval. Sections were blocked in 10% Normal Goat Serum and stained with 2 μg/mL cytokeratin 7 antibody (clone OV-TL 12/30, Dako Laboratories #M7018). Secondary and tertiary steps were undertaken using a Histostain-plus kit (Thermofisher Scientific). Colour was developed with AEC, and sections counterstained in Gills II Haematoxylin, prior to mounting with Aquamount.

### Immunofluorescence

Paraformaldehyde-fixed term human placental villi were stained with FITC-Aleuria Aurantia Lectin to visualise blood vessels, Biotin Datura Stramonium Lectin with Streptavidin 680 to visualise the trophoblast and Rhodamine-labelled Pisum sativum Agglutinin which binds the stroma (all Vector Laboratories), and imaged by confocal microscopy.

### Electron microscopy

Villous tissue from term placenta was fixed in 2.5% glutaraldehyde in 0 1 M cacodylate buffer, post-fixed in 1% osmium tetroxide and embedded in epoxy resin. Semi-thin sections were stained with 1% toluidine blue in 1% borax for examination at the light microscopic level. Ultrathin sections from suitable areas were stained with uranyl acetate and lead citrate before examining in an AEI EM6B electron microscope.

### Reporting summary

Further information on research design is available in the Nature Research Reporting Summary linked to this article.

## Supplementary information

Reporting summary

## Data Availability

All relevant data are available from the authors upon request.
