# Supplementary material for: ‘Fetal side’ of the placenta: anatomical mis-annotation of carbon particle ‘transfer’ across the human placenta
Source: Nat Commun. 2021 Dec 3;12:7049. doi: 10.1038/s41467-021-26437-y (PMC8642423; doi:10.1038/s41467-021-26437-y)
Supplement: Supplementary file 1 — Reporting summary [file 41467_2021_26437_MOESM1_ESM.pdf]

nature portfolio

Corresponding author(s): Beth Holder

Last updated by author(s): Jul 21, 2021

Reporting Summary

Nature Portfolio wishes to improve the reproducibility of the work that we publish. This form provides structure for consistency and transparency in reporting. For further information on Nature Portfolio policies, see our Editorial Policies and the Editorial Policy Checklist.

Statistics

For all statistical analyses, confirm that the following items are present in the figure legend, table legend, main text, or Methods section.

n/a	Confirmed	
☒	☐	The exact sample size (*n*) for each experimental group/condition, given as a discrete number and unit of measurement
☒	☐	A statement on whether measurements were taken from distinct samples or whether the same sample was measured repeatedly
☒	☐	The statistical test(s) used AND whether they are one- or two-sided<br>*Only common tests should be described solely by name; describe more complex techniques in the Methods section.*
☒	☐	A description of all covariates tested
☒	☐	A description of any assumptions or corrections, such as tests of normality and adjustment for multiple comparisons
☒	☐	A full description of the statistical parameters including central tendency (e.g. means) or other basic estimates (e.g. regression coefficient) AND variation (e.g. standard deviation) or associated estimates of uncertainty (e.g. confidence intervals)
☒	☐	For null hypothesis testing, the test statistic (e.g. *F*, *t*, *r*) with confidence intervals, effect sizes, degrees of freedom and *P* value noted<br>*Give P values as exact values whenever suitable.*
☒	☐	For Bayesian analysis, information on the choice of priors and Markov chain Monte Carlo settings
☒	☐	For hierarchical and complex designs, identification of the appropriate level for tests and full reporting of outcomes
☒	☐	Estimates of effect sizes (e.g. Cohen's *d*, Pearson's *r*), indicating how they were calculated

Our web collection on statistics for biologists contains articles on many of the points above.

Software and code

Policy information about availability of computer code

Data collection	NA
Data analysis	NA

For manuscripts utilizing custom algorithms or software that are central to the research but not yet described in published literature, software must be made available to editors and reviewers. We strongly encourage code deposition in a community repository (e.g. GitHub). See the Nature Portfolio guidelines for submitting code & software for further information.

Data

Policy information about availability of data

All manuscripts must include a data availability statement. This statement should provide the following information, where applicable:

- Accession codes, unique identifiers, or web links for publicly available datasets
- A description of any restrictions on data availability
- For clinical datasets or third party data, please ensure that the statement adheres to our policy

All relevant data are available from the authors upon request.

Field-specific reporting

Life sciences study design

All studies must disclose on these points even when the disclosure is negative.

Sample size	No sample size calc was performed. This is a matters arising just showing some example pictures of placenta
Data exclusions	NA. This is a matters arising just showing some example pictures of placenta
Replication	NA. This is a matters arising just showing some example pictures of placenta
Randomization	NA This is a matters arising just showing some example pictures of placenta
Blinding	NA This is a matters arising just showing some example pictures of placenta

Reporting for specific materials, systems and methods

We require information from authors about some types of materials, experimental systems and methods used in many studies. Here, indicate whether each material, system or method listed is relevant to your study. If you are not sure if a list item applies to your research, read the appropriate section before selecting a response.

Materials & experimental systems

n/a	Involved in the study	
☐	☒	Antibodies
☒	☐	Eukaryotic cell lines
☒	☐	Palaeontology and archaeology
☒	☐	Animals and other organisms
☐	☒	Human research participants
☒	☐	Clinical data
☒	☐	Dual use research of concern

Methods

n/a	Involved in the study	
☒	☐	ChIP-seq
☒	☐	Flow cytometry
☒	☐	MRI-based neuroimaging

Antibodies

Antibodies used	Sections were blocked in 10% Normal Goat Serum and stained with 2 µg/mL cytokeratin 7 antibody (clone OV-TL 12/30, Dako Laboratories #M7018). Secondary and tertiary steps were undertaken using a Histostain-plus kit (Thermofisher Scientific).
Validation	validated by the manufacturer

Human research participants

Policy information about studies involving human research participants

Population characteristics	Female pregnant.
Recruitment	Recruited during antenatal care. This is a matters arising just showing some representative images of normal placenta.
Ethics oversight	Where required, we have complied with all relevant ethical regulations, and informed consent was obtained from participants. Ethical approval was provided by the Northern X Regional Ethics Committee (New Zealand; NTX/12/06/057/AM09) or Southampton and Southwest Hampshire Local Research Ethics Committee (UK; 11/SC/0529)

Note that full information on the approval of the study protocol must also be provided in the manuscript.
